# Brainstem Encephalitis With Low-Titer Acetylcholine Receptor Antibodies Mimicking Myasthenia Gravis

**DOI:** 10.3389/fneur.2019.00829

**Published:** 2019-08-02

**Authors:** Ilya Ayzenberg, Gisa Ellrichmann, Christoph Schroeder, Lars Tönges, Anja Klasing, Vaia Pappa, Wolfgang Brück, Ralf Gold

**Affiliations:** ^1^Department of Neurology, St. Josef Hospital Bochum, Ruhr University Bochum, Bochum, Germany; ^2^Department of Neurology, Sechenov First Moscow State Medical University, Moscow, Russia; ^3^Institute of Neuropathology, University Medical Center, Göttingen, Germany

**Keywords:** brainstem encephalitis, myasthenia gravis, GAD, Ma2, sudden death

## Abstract

**Objective:** To report a rare case of brainstem encephalitis with low-titer acetylcholine receptor antibodies mimicking myasthenia gravis.

**Methods:** The patient was investigated with repeated brain MRI, CSF examination, repetitive nerve stimulation, thoracic CT, and serologic screening. Our patient passed away and finally autopsy revealed a definitive diagnosis. Written informed consent was obtained from the relatives of the patient for access to clinical files for research purposes and publication.

**Results:** We present a young woman with a subacute bulbar syndrome, who was initially diagnosed with myasthenia gravis based on clinical finding and elevated acetylcholine receptor antibodies. Episodes of numbness in the pharynx and tongue and moderate saccadic horizontal and vertical pursuits were atypical. Despite initial stabilization with intravenous immunoglobulins she developed acute asphyxia after regurgitation of food and had to be resuscitated with ultimately lethal outcome. Autopsy revealed an autoimmune T-cell mediated brainstem encephalitis. Serological screening revealed positive GAD and Ma2 autoantibodies, indicating its probable paraneoplastic nature.

**Conclusions:** Brainstem encephalitis is an important differential diagnosis even in seropositive bulbar myasthenia gravis, as several autoimmune processes often co-occur. Sudden unexpected death must be taken into account in brainstem encephalitis, requiring prolonged monitoring of the patients.

## Introduction

Co-existence of several antineuronal autoantibodies is widely known in paraneoplastic and primary autoimmune disorders. Several cases of autoimmune encephalitis and a number of paraneoplastic autoantibodies have been reported in myasthenia gravis (MG), including glutamic acid decarboxylase (GAD), voltage-gated potassium channel and collapsin response-mediator protein-5 autoantibodies (1, 2). Here we report a case of pathologically proven subacute bulbar encephalitis and low-titer acetylcholine receptor antibodies, initially misdiagnosed as a bulbar MG.

## Case-Report

A 29-years-old woman with a history of depression and cryptogenic epilepsy slowly developed progressive bulbar signs with dysarthria and dysphagia and was diagnosed with a seropositive MG due to slightly increased titer of acetylcholine receptor antibodies (0.71 nmol/l, normal < 0.4). Despite slightly increased antinuclear antibodies (1:160) there was no clinical evidence of any concomitant rheumatologic disease. Chest CT-scan revealed no malignancies or thymus hyperplasia. Surprisingly there was no improvement under pyridostigmin and the patient was referred to our hospital. Under the assumption of MG exacerbation intravenous immunoglobulin (1 g/kg) was started. In addition to dysarthria, dysphagia and bilateral facial weakness she reported episodes of slight numbness in the pharynx and tongue. She had no double vision, yet showed moderate saccadic horizontal and vertical pursuits, atypical for MG. Because of these findings polyneuritis cranialis was discussed as a possible alternative diagnosis, however anti-ganglioside antibodies were not indicative. CSF analysis revealed a discrete lymphocytic pleocytosis with 6 cells/μl by slightly increased albumin-quotient and massive local IgM synthesis (63%). Electroneurography was normal and there was no decrement in a repetitive 3/s nerve stimulation. Repeated brain MRI revealed no abnormalities. Despite initial stabilization she developed a sudden asphyxia due to regurgitation of food and bronchospasm several days later and died due to hypoxic brain injury.

**Figure 1:**
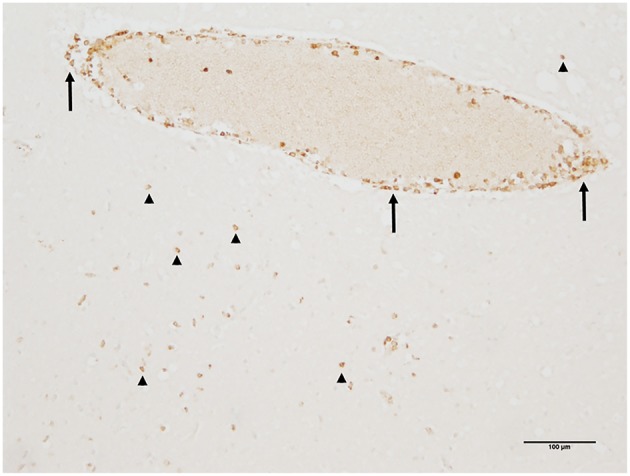
Perivascular and brain parenchymal T-cell infiltration in the midbrain. Immunohistochemistry identified these inflammatory cells as CD3-positive T cells being located mainly perivascularly (arrows) but also invading the brain parenchyma (arrowheads) (immunohistochemistry for CD3).

Autopsy revealed extensive hypoxic damage in all gray matter areas of the brain with neuronal necrosis and already infiltrating granulocytes. Within the brain stem, especially in midbrain and pons, perivascular lymphocytic infiltrates were seen. Immunohistochemical investigation identified these inflammatory cells as CD3-positive T cells, located perivascularly as well as within the brain parenchyma (Figure 1). B cells or plasma cells were not detected. Screening for the antineuronal autoantibodies revealed positive GAD and Ma2 autoantibodies in serum. However, no thymoma or other tumors have been detected on autopsy despite of extensive examination of inner organs.

## Discussion

An autoimmune brainstem encephalitis was the core pathology in our patient initially misdiagnosed as MG. The level of AChR antibodies was very low and had to be interpreted with caution. Although an additional pathogenetic role of the AChR antibodies cannot be definitely excluded, non-responsiveness to pyridostigmin and absence of a decremental response in a repetitive stimulation make it rather unlikely. Additional clinical signs, such as saccadic pursuits and sensory deficits, even if mild, are also atypical for MG. The disruption of the blood-brain-barrier and massive intrathecal IgM production confirmed an inflammatory CNS process. Immunohistopathology revealed a T-cell mediated inflammation in brainstem, typical for a paraneoplastic encephalitis, associated with autoantibodies against intracellular antigens.

Despite positive GAD- and Ma2-antibodies no tumor has been found. Patients with anti-Ma2 antibodies often develop paraneoplastic limbic or brainstem encephalitis at a very early tumor stage, including carcinoma *in situ* (3). Interestingly, eye movement abnormalities are characteristic and have been seen in 92% of those with brainstem involvement. Anti-GAD65 autoantibodies are in many cases non-paraneoplastic, however the probability of underlying cancer (mostly thymoma, lung or breast cancer) is 7-fold higher if further antineuronal antibodies coexist (4). Yet positive antinuclear antibodies and young age of our patient support probable primary autoimmune origin. Laryngospamus, respiratory or cardiac arrest have been reported as possible reasons of sudden death in brainstem encephalitis (5).

## Conclusion

We present a patient with an autoimmune brainstem encephalitis and multiple autoantibodies, mimicking a bulbar form of MG. Even if mild, atypical clinical signs including sensory and eye movement abnormalities, should raise concerns about an exclusive diagnosis of MG. Sudden unexpected death must be taken into account in patients with brainstem encephalitis, requiring prolonged monitoring during disease deterioration.

## Data Availability

The raw data supporting the conclusions of this manuscript will be made available by the authors, without undue reservation, to any qualified researcher.

## Ethics Statement

Written patient consent was obtained for publication of this case report.

## Author Contributions

IA: acquisition, analysis and interpretation of data, critical revision of the manuscript for important intellectual content. GE, AK, and VP: acquisition of data, critical revision of the manuscript for important intellectual content. CS and LT: acquisistion of data, critical revision of the manuscript for important intellectual content. WB: analysis and interpretation of data, critical revision of the manuscript for important intellectual content. RG: study concept and supervision, critical revision of the manuscript for important intellectual content.

### Conflict of Interest Statement

IA received board honoraria from Merck Serono, Roche, travel grant from Biogen Idec and grant support from Chugai Pharma. GE received speakers honoraria of BogenIdec, TEVA Pharma, Bayer Healthcare, Genzyme, Almirall and Novartis Pharma. She received scientific grant form BiogenIdec, TEVA Pharma and Novartis Pharma. LT received travel funding and/or speaker honoraria from Abbvie, Bayer, Bial, Desitin, GE, UCB, Zambon and consulted for Abbvie, Bayer, Bial, Desitin, UCB, Zambon. RG received speaker's and board honoraria from Baxter, Bayer Schering, Biogen Idec, CLB Behring, Genzyme, Merck Serono, Novartis, Stendhal, Talecris and TEVA. His department received grant support from Bayer Schering, BiogenIdec, Genzyme, Merck Serono, Novartis and TEVA. The remaining authors declare that the research was conducted in the absence of any commercial or financial relationships that could be construed as a potential conflict of interest.
